# Microbial Ecology of the Hive and Pollination Landscape: Bacterial Associates from Floral Nectar, the Alimentary Tract and Stored Food of Honey Bees (*Apis mellifera*)

**DOI:** 10.1371/journal.pone.0083125

**Published:** 2013-12-17

**Authors:** Kirk E. Anderson, Timothy H. Sheehan, Brendon M. Mott, Patrick Maes, Lucy Snyder, Melissa R. Schwan, Alexander Walton, Beryl M. Jones, Vanessa Corby-Harris

**Affiliations:** 1 Center for Insect Science, University of Arizona, Tucson, Arizona, United States of America; 2 USDA-ARS Carl Hayden Bee Research Center, Tucson, Arizona, United States of America; 3 Department of Microbiology, University of Arizona, Tucson, Arizona, United States of America; 4 School of Life Sciences, Arizona State University, Tempe, Arizona, United States of America; 5 Department of Ecology and Evolution, Iowa State University, Ames, Iowa, United States of America; 6 Department of Entomology, University of Illinois, Urbana-Champaign, Illinois, United States of America; Emory University, United States of America

## Abstract

Nearly all eukaryotes are host to beneficial or benign bacteria in their gut lumen, either vertically inherited, or acquired from the environment. While bacteria core to the honey bee gut are becoming evident, the influence of the hive and pollination environment on honey bee microbial health is largely unexplored. Here we compare bacteria from floral nectar in the immediate pollination environment, different segments of the honey bee (*Apis mellifera*) alimentary tract, and food stored in the hive (honey and packed pollen or “beebread”). We used cultivation and sequencing to explore bacterial communities in all sample types, coupled with culture-independent analysis of beebread. We compare our results from the alimentary tract with both culture-dependent and culture-independent analyses from previous studies. Culturing the foregut (crop), midgut and hindgut with standard media produced many identical or highly similar 16S rDNA sequences found with 16S rDNA clone libraries and next generation sequencing of 16S rDNA amplicons. Despite extensive culturing with identical media, our results do not support the core crop bacterial community hypothesized by recent studies. We cultured a wide variety of bacterial strains from 6 of 7 phylogenetic groups considered core to the honey bee hindgut. Our results reveal that many bacteria prevalent in beebread and the crop are also found in floral nectar, suggesting frequent horizontal transmission. From beebread we uncovered a variety of bacterial phylotypes, including many possible pathogens and food spoilage organisms, and potentially beneficial bacteria including *Lactobacillus kunkeei*, Acetobacteraceae and many different groups of Actinobacteria. Contributions of these bacteria to colony health may include general hygiene, fungal and pathogen inhibition and beebread preservation. Our results are important for understanding the contribution to pollinator health of both environmentally vectored and core microbiota, and the identification of factors that may affect bacterial detection and transmission, colony food storage and disease susceptibility.

## Introduction

Much of the world's agricultural needs rely on pollination by honey bees, but like other pollinators, honey bee populations have declined steadily for many years. The vast numbers of honey bees needed for adequate pollination are maintained primarily with the prophylactic application of antibiotics, supplemental feeding and biocides to control parasites. During this recent decline, research has tended towards understanding stress or disease phenotypes resulting from the interaction of pathogens or some combination of factors that might compromise the colony [1]–[4]. More recently, work has begun in earnest to define the contribution of beneficial and seemingly benign microbes to both colony and ecosystem health [5].

The honey bee is a generalist pollinator that fills its living quarters with nutritionally rich resources. While the core bacterial microbiota of the adult honey bee gut is composed of only a handful of major taxonomic groups, moderately abundant and/or transient bacteria found throughout the hive environment are highly diverse [5]–[8]. From a microbial perspective, the honey bee consists of individual, group, and hive components, complete with a large repertoire of homeostatic behaviors that regulate or influence temperature, moisture, osmotic potential, ambient exposure, and pH. Consequently, this combination of group dynamics and hive physiology has been labeled a superorganism [9], [10]. As a complex homeostatic system, the honey bee and its ever changing hive presents a broad spectrum of microenvironments with the potential for rigid to highly diffuse microbial fidelity [5]. The variety of pollination environments and abundance of micro-niches within the honey bee hive provide a unique setting for revealing the roles of microbial community assembly in host health.

Symbiotic associations between insects and microorganisms are common, with nutritional contributions from obligate intracellular endosymbionts receiving the most research attention [11]–[13]. The near ubiquitous nature of extracellular gut symbionts in insects and other arthropods is widely accepted, but there has been very little attention paid to their roles [11], [14]. Nonetheless, many recent studies have sought to define core gut bacterial communities as a platform to investigate coevolution and host-microbial ecology [7], [8], [15], [16]. In animals, gut bacteria can provide nutrition or protection from pathogens, be vertically transmitted and obligate for host survival, or environmentally vectored yet still contribute significantly to host fitness [11], [17]–[19]. While some gut bacteria are highly specialized, interfacing with host metabolism and tissue on a molecular level, others retain the ability to occupy a multitude of niches distinct from the host gut [17], [20], [21]. Such environmentally acquired microbes can be facultative symbionts, showing limited if any host specificity, with their presence/abundance often dependent on host development or health, geography or environmental conditions [12], [22].

There are two present paradigms for the interpretation of core gut bacteria in honey bees, one focused on the crop (fore-gut, honey stomach or social stomach), and the other emphasizing the gut exclusive of the crop (Table 1). The presently accepted core gut microbiota of the honey bee is composed of 7–12 regularly occurring phylotypes that constitute 99% of the sequences found in the gut of a bee with deep sequencing. Some of these symbionts may be found in the midgut, but most seem to occur primarily in the rectum and/or ileum as a biofilm [7], [8], [19], [23]–[27]. For the purposes of this paper we define the core gut bacteria as that assemblage of phylotypes found in 80–100% of sampled bee guts revealed by 454-amplicon sequencing of both active (rRNA) and present (rDNA) bacteria [7], [8], [27]. As the majority of this sequence data was derived from the midgut and hindgut exclusive of the crop, the term “gut” will henceforth refer to the ventriculus, ileum and rectum.

**Table 1:** Bacteria considered core to the crop or gut of the honey bee.

Clade labels*	Related genera or species	Crop specific strains (cultured isolates)†		Honey bee 16S clone (top megaBLAST hit)		
		Strain name	Accession number	Nucleotide similarity	Accession number	Tissue¶
Bifido	*Bifidobacterium* sp.	Bin2	EF187231	1357/1369	AY370184	SA
Bifido	*Bifidobacterium* sp.	Bin7	EF187234	1325/1325	HM113212	PG
Bifido	*Bifidobacterium* sp.	Hma3	EF187236	1362/1369	AY370184	SA
Bifido	*Bifidobacterium* sp.	Bma6	EF187237	1320/1324	HM112025	SA
Firm 4	*Lactobacillus* sp.	Hon2	EF187244	1364/1367	HM111964	SA
Firm 4	*Lactobacillus* sp.	Bin4	EF187245	1362/1364	HM112042	SA
Firm 5	*Lactobacillus* sp.	Hma8	EF187243	1395/1410	AY370183	SA
Firm 5	*Lactobacillus* sp.	Hma11	EU753689	830/830	HM113344	PG
Firm 5	*Lactobacillus* sp.	Bma5	EF187242	1368/1368	HM111880	SA
Firm 5	*Lactobacillus* sp.	Hma2	EF187240	1401/1410	HM046569	MG
Firm 5	*Lactobacillus* sp.	Biut2	EF187240	1398/1411	HM046569	MG
n/a	*Lactobacillus kunkeei*	Fhon2	EF187239	1416/1419	HM008721	MG
n/a	*Lactobacillus kunkeei*	Fhon13	HM534758	1426/1446	HM008721	MG
Consistently found in the mid/hind gut §						
Alpha 2.1	*Commensalibacter* sp.	n/a	EU409601	n/a	n/a	n/a
Alpha 2.2	*Saccharibacter* sp.	n/a	AJ971850	n/a	n/a	n/a
Beta	*Snodgrassella alvi* ‡	n/a	DQ837617	n/a	n/a	n/a
Gamma 1	*Gilliamella apicola* ‡	n/a	AY370191	n/a	n/a	n/a
Gamma 2	*Frischella perrara* ‡	n/a	HM108316	n/a	n/a	n/a

*Labels and putative members of the core gut microbiota according to phlogenetic clade membership [8], [19], [25]–[27], [38].

† Thirteen bacterial isolates hypothesized to represent the core microbiota of the crop [29]–[33].

¶ Single abdomen; SA, 80 pooled guts; PG, mid gut; MG.

§ *Bifidobacterium*, *Lactobacillus* Firm4 and Firm5 are also found consistently in the mid/hind gut [7], [8], [24], [44], [45].

‡ Recently named and described [7], [38], [55].

The foregut or crop is the honey bee's nutritional interface with the pollination environment, colony food stores and other colony members. This portion of the alimentary tract is not involved in digestion, but is essentially an inflatable storage bag designed to transport nectar from the flower to the hive and to share liquid nutrition with sibling nestmates [28]. Pollen foragers mix collected pollen with crop contents prior to consolidation in hind leg corbicular baskets. The collected pollen pellets are packed into wax storage cells within the hive, and become beebread, a hive storage product rich in microbes, vitamins, lipids and proteins. Hence the crop has been described as part of a widespread microbial niche that may include beebread, honey, floral nectar, and the phyllosphere [5]. The crop is also considered an extreme environment harboring very few bacteria, subject to behavioral activities, pH and enzymes that discourage biofilm formation [5], [7]. A recent series of papers advocates that 13 particular strains of bacteria comprise the core microbiota of the crop (Table 1), as distinguished from bacteria that preferentially occupy the gut [29]–[33]. It is hypothesized that these 13 crop bacteria work synergistically to protect the honey bee and its food stores from detrimental microbes. While the core gut bacteria are readily detected, inconsistent detection of all 13 bacterial strains from the crop niche has been attributed to sampling depth or time, host health, environmental conditions, or food sources [33]. The results however, are based solely on sequencing the 16S rDNA of cultured isolates from the crop, making it difficult to know if these 13 bacterial strains are the result of culturing bias, are specific to the crop, or occur in other gut compartments or microenvironments.

Our general goal is to capture, describe, and compare bacterial diversity according to sampled niche and methodology. We rely strongly on culturing to confirm previous work, and demonstrate the viability of bacteria retrieved from various niches. We hypothesize that many bacteria commonly found in the crop are transient and specific to the hindgut, or that stored food and other hive environments represent a secondary niche for hindgut bacteria, or those vectored from the phyllosphere or floral nectar. Specifically we address 1) the culturability and diversity of bacteria from floral nectar, different segments of the honey bee alimentary tract, honey and stored pollen provisions, 2) the evolutionary relationships, potential niche breadth, and transmission potential of honey bee related bacteria, and 3) the hypothesis that there is a core microbiota specific to the crop. Studies on the crop have used primarily *Lactobacillus* specific (MRS) media to culture viable bacteria [29]–[33]. Here we use both clones and isolates cultured from a variety of different growth media. Because 454-amplicons (pyrotag data) are typically too short to evaluate strain variation, we acquire near full length sequences of the 16S rRNA gene. Armed with the knowledge that cultivation and molecular-based techniques are each subject to their own particular methodological bias, we use a comparative approach to more thoroughly characterize microbial diversity.

## Materials and Methods

### Ethics Statement

All colonies were sampled from apiaries located at the USDA Carl Hayden Bee Research Center in Tucson AZ. Our field collections did not involve endangered or protected species, and no specific permissions were required, because the study was conducted by USDA employees.

### Bacterial culturing and isolation

We cultured bacteria from nine different honey bee (*Apis mellifera*) associated microenvironments, including honey, beebread, three distinct regions of the alimentary tract, and four different species of floral nectar from flowers from the immediate pollination environment (Table S1). The five media used to culture the majority of samples were: de Man Rogosa Sharpe (MRS), favoring the growth of Lactobacilliales, Sabaroud dextrose agar (SDA) and Candida agar, both low pH fungal media, and two general-purpose media with near neutral pH; Brain-heart infusion (BHI) and Tryptic soy agar (TSA). To attain bacterial isolates, samples were processed according to microenvironment (see below), and approximately 10 ul of this solution was streaked onto the media with an inoculation loop under sterile conditions in a laminar flow biological cabinet. Sample size by colony, plant and gut microenvironment can be found in Table S1. See (Text S1) for culturing details.

Bacteria from all microenvironments were cultured in the dark at 35 C, the optimal temperature of the hive [9]. After 3–5 days, bacterial isolates were picked randomly, re-streaked and subcultured from one to several times in 4 ml of their respective media broth. Samples with observed growth were vortexed to suspend bacterial cells, and a 1 ml aliquot was transferred to 1.5 ml tubes and centrifuged (12,000 g) for 5 min. Growth media was decanted and the pelleted cells were processed for gram positive DNA extraction using the Fermentas GeneJet Genomic DNA Purification Kit following the protocol for gram-positive bacteria. We PCR amplified the 16S rRNA gene according to established protocols [16]. The resulting PCR product was sequenced in both directions using an Applied Biosystems 3730 DNA Analyzer at the Bio5 institute in Tucson, AZ. Universal PCR primers are listed in the supplementary files (Tables S2–S5).

### Beebread bacterial clones

Because previous culture dependent work revealed that beebread was microbially rich [6], we coupled our culturing effort with culture-independent methods to reveal the hidden bacterial diversity of beebread. One clone library was built from each of two neighboring colonies that had ceased brood rearing and were storing pollen for overwintering (Table S4). We sampled beebread with sterile aluminum tubes by coring individual cells on a frame. We homogenized samples by vortexing. Immediately after vortexing we removed a 200 µl aliquot of suspended pollen to a new 1.5 ml centrifuge tube. We added 300 µl of TE/Triton Buffer (20 mM Tris-HCL, 2 mM EDTA, 1.2% Triton X-100, pH 8.0) and vortexed for 5 minutes. Each sample was briefly centrifuged and the supernatant was removed to a new tube. The above wash cycle was repeated 4 times and the resulting volume of supernatant (1.4 ml) was centrifuged for 30 minutes on high to pellet the bacterial cells. Total DNA was isolated and purified using the Fermentas GeneJet Genomic DNA Purification Kit following the protocol for gram-positive bacteria, which included the addition of lysozyme 20 mg/ml to TE/Triton buffer followed by 30 minute incubation at 37°C. We PCR amplified 16SrRNA genes from bacterial communities using universal primers 27F and 1391R according to established protocols [16]. PCR products were cloned using the Invitrogen TopoTA system (vector pCR2.1), using One Shot (Invitrogen, Carlsbad, CA, USA) chemically competent *E. coli* cells for transformation and blue-white colony screening on LB plates with kanamycin. White colonies were picked and grown overnight in LB media. We amplified the cloned 16S rRNA fragments from the TopoTA plasmid using vector primers M13F and M13R and sequenced the resulting PCR product in both directions using the M13F/R primer pair.

### 16S sequence processing and taxonomy

All 16S rRNA gene sequences were assembled and edited with Bioedit [34], and the remaining vector sequence was removed from cloned sequences. Chimeric sequences were eliminated with Bellerophon (version 3) [35] on the Greengenes website [36], and results were manually rechecked. Sequences were then uploaded, aligned and classified on the Ribosomal Database Project website [37] and queried against NCBI's database using BLASTn and megaBLAST. We removed all sequences returned as chloroplast DNA, and retained 1723 sequences of the 16S rRNA gene for further phylogenetic or comparative analyses. Sequences were deposited in GenBank under accession numbers KF598867–KF600589.

### Core bacteria comparisons

Acid tolerant bacteria from the crop and food stores have been cultured using *Lactobacillus* targeted (MRS) and other acidic media, and members of the core gut bacterial community have also been cultured [33], [38]. To determine the general utility of cultivation for revealing core gut bacteria, we compare our findings to culture-dependent and culture-independent assessments from previous studies of the alimentary tract.

Published results are conflicting concerning the existence of a crop biofilm, the number of bacteria in the crop, and the mode by which newly emerged bees acquire the core bacteria [7], [33]. To evaluate the hypothesis of a core crop microbiota, we first attempted to amplify bacterial DNA directly from individual crops using universal bacterial primers. Initial attempts produced negligible template DNA and inconsistent PCR products. These results agreed with Martinson et al. [7], who found only 10^4^ bacterial 16S rDNA gene copies in the crop using qRT-PCR. Thus, we took an approach similar to previous studies [29]–[33], and cultured deeply from the crop using largely MRS media to select for acid tolerant bacteria like *Lactobacillus*. Because the core gut bacteria have been identified by culture-independent methods [8], we cultured from the mid and hindgut to capture the viable core diversity, and assess potential culturing bias (Table S2). To further address the impact of culturing bias, we compare taxon (genera) richness between basic and acidic media by performing a rarefaction analysis in EcoSim [39]. Rarefaction curves were calculated with 100 iterations and sampling without replacement.

To determine whether the 16S sequence can be used as a marker to distinguish the bacteria in the crop from other alimentary tract microenvironments, we compared our sequenced crop and hindgut isolates (Table S3) to GenBank 16S sequences derived from other studies that performed culture-dependent assessments of the crop, culture-independent assessments (16S cloning) of the entire alimentary tract or abdomen, the gut exclusive of the crop, and pooled gut samples. This comparison conservatively allowed for 0.1% (1 in 1000 bp) difference due to sequencing error, base-calling or 16S assembly. A preliminary GenBank survey of the 13 putative core crop strains [29]–[33], indicates that only 3 of 13 near full length 16S sequences are identical to cloned sequences found in other studies of honey bee microbiota (Table 1).

### Microbial transmission

Earlier attempts to uncover the core bacteria from newly emerged bees and beebread using culture-independent methods revealed few to no bacteria corresponding to the characteristic gut phylotypes [7]. It has been hypothesized that putative core crop *Bifidobacterium* and *Lactobacillus* build up gradually by trophallactic exchange with nestmates [33]. To investigate the potential for microbial transmission via the hive environment, we cultured bacteria from food stores and the crops of newly emerged bees (NEB's) denied trophallactic contact with older siblings, but given access to beebread and honey. Wax comb containing capped and emerging brood, capped and uncapped honey, and beebread were removed from their parent colonies, shaken clean of adult worker bees, and placed in an enclosed incubator at 35°C and 50% relative humidity. New worker bees were allowed to emerge overnight and were sampled the following morning. Crops were cultured and sequenced as detailed above. The bacterial communities of NEB's were compared to those found in the crops of random in-hive bees (IHB's). We compared only those crop isolates derived from MRS media using a chi-squared test in EcoSim [39].

### Comparative analyses of community structure

Although species concepts are difficult to apply to bacteria, <3% sequence divergence is considered standard for grouping bacteria into Operational Taxonomic Units (OTU's). Presently, the bacterial phylotypes considered core to the honey bee gut are grouped according to monophyly (Table 1), with members of a single clade often exceeding 3% sequence divergence depending on the compared size and region of the 16S rRNA gene [26]. Given the present understanding of the system, we adopt this phylogenetic definition for some comparative purposes in this paper.

We placed our bacterial survey in the context of culture-independent results by designing a simulated core gut bacterial community consisting of those phylogenetic groups occurring in 80–100% of individual bee guts according to 454-amplicon pyrosequencing from three remote locations [8], [27]. We first determined whether the Acetobacteraceae (Alpha 2) phylotype reported by these manuscripts corresponded to the Alpha 2.1 or Alpha 2.2 phylogenetic clade [26]. From the GenBank Sequence Read Archive we downloaded and analyzed four published amplicon libraries (accession SRA046735, 16S gene regions V6–V8) from [8], that showed the greatest abundance of Alpha 2 phylotype from two different locations, Maryland (MD) and Arizona (AZ). Individual gut libraries according to location/colony/individual were: MD/019/3, MD/299/2, AZ/107/4, and AZ/125/4. The crops were removed from these bees prior to DNA isolation such that these sequences represent only the gut [8]. We also downloaded and analyzed the entire gut specific dataset from [40], an amplicon library (accession DRX001333, variable regions V1–V2) based on rRNA converted to cDNA from bees in Massachusetts. This data set was recently reanalyzed using a training set that included honey bee-specific sequences [27], [41]. From these bees the entire alimentary tract (crop and gut) was used for rRNA extractions [40]. To accurately classify these amplicons we added a set of near full length honey bee-specific 16S rRNA sequences [8], [24], [26], [42], [43] to the RDP default training set. From the DRR001870 archive, all sequences were classified as Alpha 2.1 (n = 1060 reads with >97% identity). From the DRX001333 archive, 98% of the sequences were classified as Alpha 2.1 and 2% were Alpha 2.2 (n = 1392 reads with >97% identity).

Group proportions of the simulated bacterial community were 34% *Lactobacillus* Firm5, 31.5% *Gilliamella apicola* (Gamma 1), 17.5% *Lactobacillus* Firm4, 8.2% *Snodgrassella alvi*, (Beta) 5.7% *Bifidobacteria* sp., 2.1% *Frischella perrara* (Gamma 2), and 1% Acetobacteriaceae (Alpha 2.1). Bacteria occurring in 0–35% of individuals or at very low abundance were Alpha 1, Alpha 2.2, Gamma 3 and Gamma 4, and were not considered core gut bacteria for this analysis. Simulated gut communities were constructed by a random re-sampling of each 97% OTU cluster composed of full length 16S clone sequences from the mid and hindgut [24], [26], [44], [45]. To visualize relationships among the bacterial communities inhabiting each sampled microenvironment in this study, all sequences were aligned and used to reconstruct a phylogenetic tree by implementing the relaxed neighbor-joining algorithm in Clearcut [46]. The resulting tree was used for subsequent analyses on Fast UniFrac [47], a program that compares bacterial communities by assessing the fraction of phylogenetic branch length that is unique to each. Distances generated according to the unweighted Unifrac metric were exported for Principle Coordinates Analysis (PCoA) to visually explore variation among the nine sampled microenvironments and simulated gut community. We chose a nonabundance-based metric because microenvironments were subject to different sampling efforts. Therefore, community differences were measured only as bacterial taxa present or absent among samples. As a complementary beta diversity measure, we report pair wise Bray-Curtis dissimilarity indices on 97% OTUs according to raw abundance, log abundance, normalized abundance (ratios) and presence/absence of OTU's.

### Comparing isolates to clones

We compared culturing and cloning for both the gut and beebread samples by grouping sequences as 97% and 99% OTUs and determining the number of shared OTU's and proportion of shared sequences. To compare our sequenced isolates from gut cultures to clone libraries, we surveyed GenBank and downloaded near full-length 16S clone sequences derived from the midgut [24], [44], and from both the midgut and hindgut [8], [45]. Both clone and isolate sequences were aligned against the Silva 16S rDNA SEED database [48]. Pairwise distance matrices were constructed separately for the bee bread and gut libraries and sequences were assigned to operational taxonomic units (OTUs) based on 97% and 99% levels of sequence similarity using the furthest neighbor algorithm. Venn diagrams were constructed to determine the number of OTUs shared between cloned and isolated samples for each library. Sequence data were analyzed using Mothur version 1.26.0 [49].

Beebread clones and isolates, both generated by this study, were compared as described above for shared OTU's. The structure of cloned bacterial communities from beebread was compared between the two sampled colonies using UniFrac (unweighted). We also report alpha diversity metrics and rarefaction results for beebread bacterial communities derived from both sequenced clones and isolates. The community was rarefied before analyses by re-sampling 97% OTUs without replacement. We determined Good's coverage, Shannon and Simpson indices for each sample.

### Phylogenetic analyses

To provide insight into the diversity, evolutionary context and niche specificity of the identified microbes, we performed a phylogenetic analysis for the three most abundant groups of microbes in the crop and beebread; the Firmicutes, Actinomycetales, and Alpha 2.2 (Acetobacteraceae). We utilized results from the RDPII naïve Bayesian classifier to collate taxon-specific datasets [50]. For both groups, identical or closely related 16S rRNA sequences were retrieved from GenBank using the megaBLAST algorithm. We assessed sequence variation using DNAsp software [51] to estimate average pairwise sequence divergence for all core gut bacteria, and minimum number of recombination events for novel sequences in all three phylogenetic datasets (Table S6). Multiple sequence alignments were performed with CLUSTAL, and manually edited in BioEdit [34]. Phylogenetic analyses were conducted using MEGA version 4.0.2 [52]. Relationships were inferred using the Neighbor-Joining algorithm [53] according to the Maximum Composite Likelihood method with 1,000 bootstraps [54]. All positions containing gaps and missing data were eliminated from the dataset using the complete deletion option. We mapped sampled niche onto the Firmicute and Alpha 2.2 phylogenies. *Clostridia*, *Bifidobacterium*, and *Gluconobacter morbifer* served as outgroups for Firmicutes, Actinomycetales and Alpha 2.2 (Acetobacteraceae) respectively.

## Results

### Bacteria from the gut, hive and pollination environment

**Figure 1:**
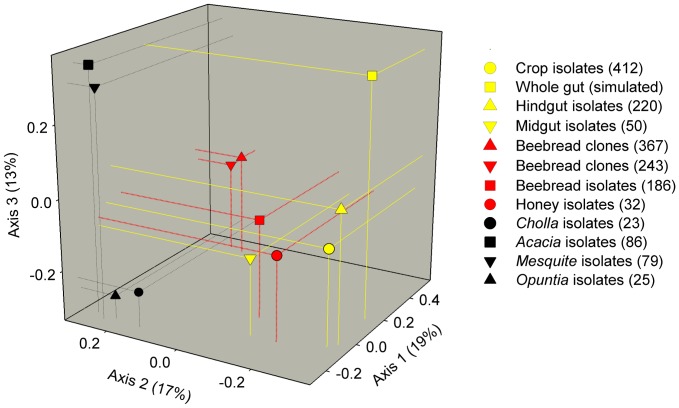
PCoA analysis of bacterial communities from *Apis mellifera* associated niches. Analysis based on unweighted UniFrac distances. Positions of the bacterial communities for each sampled niche along the first three principal coordinate axes are illustrated, along with the percentage of variation explained by each axis. The simulated gut community was composed of cloned sequences specific to the midgut and hindgut. Symbols are colored by general niche; yellow: alimentary tract, red: food stores and black: flowers. Sample size shown in parentheses.

Our efforts produced 1723 informative 16S rDNA sequences ranging between 500–1300 bp with mean sequence length >1000 bp. With the inclusion of the simulated core gut data set, our first 3 PCoA axes explained 49% of the variation in the total model (Fig. 1). The unweighted and distance-based PCoA ordination resulted in five clusters, four well-defined and one loosely-defined. To gain a sense of ordination space, the most well-defined cluster is composed of two cloned beebread samples from neighboring colonies which differed significantly according to unweighted UniFrac score (p<0.001). The loosely-defined cluster is composed of isolates from three alimentary tract and two food store environments. The simulated gut formed its own group, with the hind gut isolates as its closest neighbor. Floral environments clustered separately and according to taxonomic identity, producing a Fabaceae and Cactaceae cluster separated primarily along the third axis. Bray-Curtis estimates based on both raw and normalized abundance of 97% OTU's reveal that the greatest pairwise similarity between microenvironments occurs among beebread clone libraries, the crop and hind-gut.

**Figure 2:**
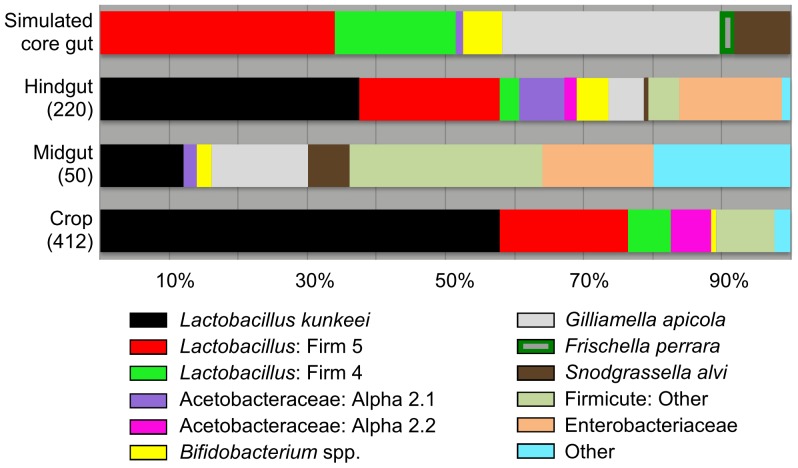
Bacterial communities in the alimentary tract. Results based on sequenced isolates from multiple growth media. Number of sequences for each niche is shown in parentheses. See methods for the determination of the simulated core gut community, and Tables S2 and S3 for detailed taxonomy.

**Figure 3:**
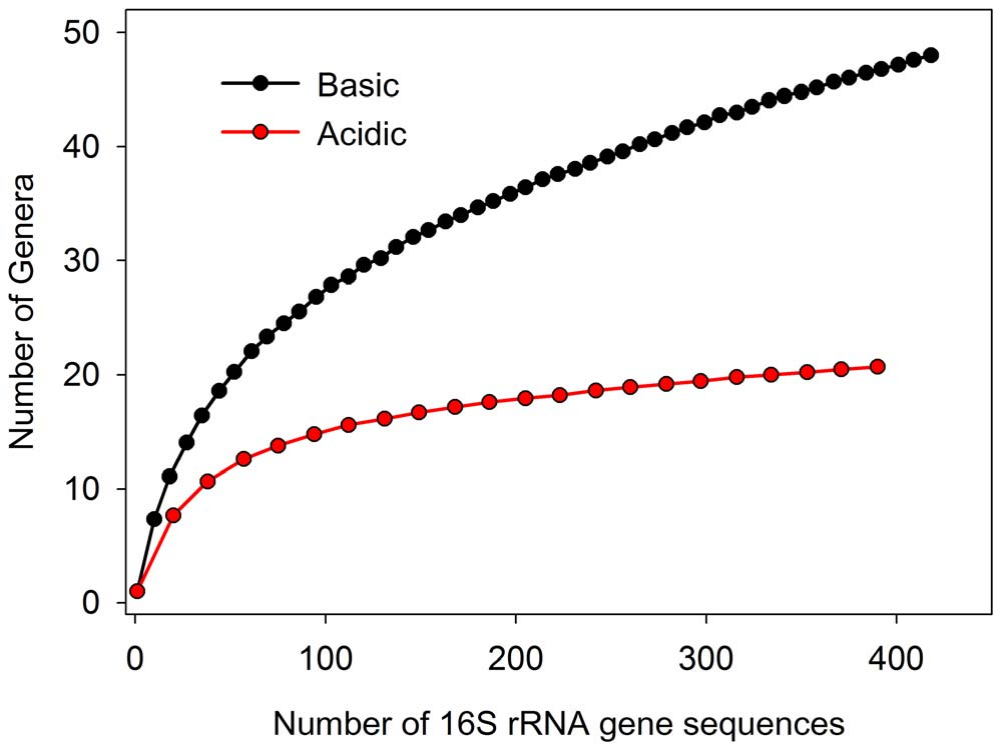
Rarefaction curves according to media pH. Genera as determined by the RDPII classifier were regarded as OTU's. Acidic media ranged in pH from 5.6–6.1, and neutral media from 7.0–7.4.

With the exception of the recently described *Frischella perrara* [55], we cultured members from all seven bacterial groups we designated as “core” to the honey bee gut (Fig. 2). At least one phylotype corresponding to each of these six core gut bacterial groups grew in the presence of oxygen (Table S1). We isolated 273 total sequences corresponding to the core gut microbiota, and more than half of these were *Lactobacillus* Firm5 (Table S6). Frequencies of *Lactobacillus* Firm5 and Firm4 were similar between crop and hindgut isolates, primarily due to the high frequency of Firm5 isolates found in the crops of NEB's (Table S7). *Lactobacillus* Firm5 and Firm4 were identified only from acidic media, primarily MRS (Table S1). *Bifidobacterium* were infrequent in the crop, but found more frequently in the hindgut when cultivated with neutral media (Table S1). Relative to the crop, the midgut and hindgut held much greater overall core and non-core diversity. *Gilliamella apicola* was isolated only from the midgut and hindgut. In agreement with their scarcity in acidic hive niches, the core gut bacteria *Gilliamella apicola*, *Snodgrassella alvi*, Alpha 2.1 (Acetobacteraceae), and *Bifidobacterium* were isolated almost exclusively from pH neutral media. While found in four different microenvironments, only six sequences corresponding to *Snodgrassella alvi* were identified among all isolates (Table S6). Also preferring pH neutral media were a variety of “non-core” gut bacteria broadly classified as Firmicutes and Enterobacteriaceae. When considering all environments, acidic media revealed the lowest diversity, while pH neutral media revealed much greater diversity, and very different communities of bacteria (Fig. 3, Table S1).

The cultivable diversity of some gut residents is a strong reflection of the taxonomy and diversity found with non-culture based methods (Fig. 1). Average sequence divergence revealed *Lactobacillus* Firm5 to have the highest cultivable diversity (Table S6). There was a significant positive relationship between the frequency with which the core bacteria were cultured and their pairwise divergence estimates (Adj Rsqr  = 0.68, F_1,6_ = 15.7, P = 0.007), indicating that more frequently cultured core groups revealed more sequence diversity (e.g. Firm5 and Alpha 2.2). Compared to the core microbiota as a whole, Firm4 and Alpha 2.1 showed low sequence divergence relative to isolate abundance, while *Bifidobacterium* showed the opposite pattern (Table S6).

Members of the core gut bacterial community were found sporadically in beebread. We cultured one isolate each of *Lactobacillus* Firm4, Firm5, and *Snodgrassella alvi*, and two isolates corresponding to Alpha 2.1 (Acetobacteraceae). In contrast, Alpha 2.2 was the most frequent isolate from honey and the second most abundant isolate in beebread (Table S1). Alpha 2.2 grew in five different media across a range of pH (5.6–7.4). Other non-core Firmicutes and Actinobacteria were also cultured from both honey and beebread.

### Putative core crop strains

The substantial use of MRS in culturing crop bacteria revealed only 2 phylotypes corresponding to the 13 putative core crop strains [29]–[33]. Of these thirteen, we found sequences corresponding to strain Fhon2; *Lactobacillus kunkeei*, and strain Bma5; *Lactobacillus* Firm5 (see Table 1). However, strain Bma5 was also the most abundant isolate from hindguts of random in-hive bees (IHB's), and was not found among the IHB crop isolates. Interestingly, the remainder of our *Lactobacillus* Firm5, Firm4 and *Bifidobacterium* crop and hindgut isolates shared ≥99.9% sequence similarity with GenBank crop isolates sampled from other bee species or genera from other continents (*Apis florea*, *Apis cerana*, and *Meliponula bocandeei*). *Lactobacillus kunkeei* was the most frequent isolate from both the crop and hindgut environment, but also found in the midgut, pure honey, floral nectar, and beebread (Table S1). *L. kunkeei* occurred year-round and grew on all tested media under both aerobic and microaerophilic conditions. To summarize, we isolated many sequences from the hindgut corresponding to putative crop-specific isolates, and many isolates from the crop corresponding to cloned sequences from the midgut or hindgut only, the entire gut or abdomen and/or 454-amplicon sequences from alimentary tract exclusive of the crop (Table S7).

### Bacterial transmission

Concerning the suitability of nectar as an environmental refuge or reservoir, isolates from four flowers in the immediate pollination environment revealed a variety of bacterial families (Fig. S1, Table S5). Collectively, all flowers were host to a variety of Enterobacteriaceae and Firmicutes. *Weissella* spp. (Leuconostocaceae), were frequent in both of the sampled cacti (*Opuntia* and *Cholla* spp.), while *Bacillus* spp. were more often cultivated from species of *Acacia* and *Mesquite*. Sequences of five isolates from *Mesquite* flowers were identified as *Lactobacillus* Firm5, and three of the four sampled flowers held viable *L. kunkeei*. Considering all flower environments, 38 of 215 (17.7%) sequenced isolates were identical to honey bee gut, crop, or hive samples.

**Figure 4:**
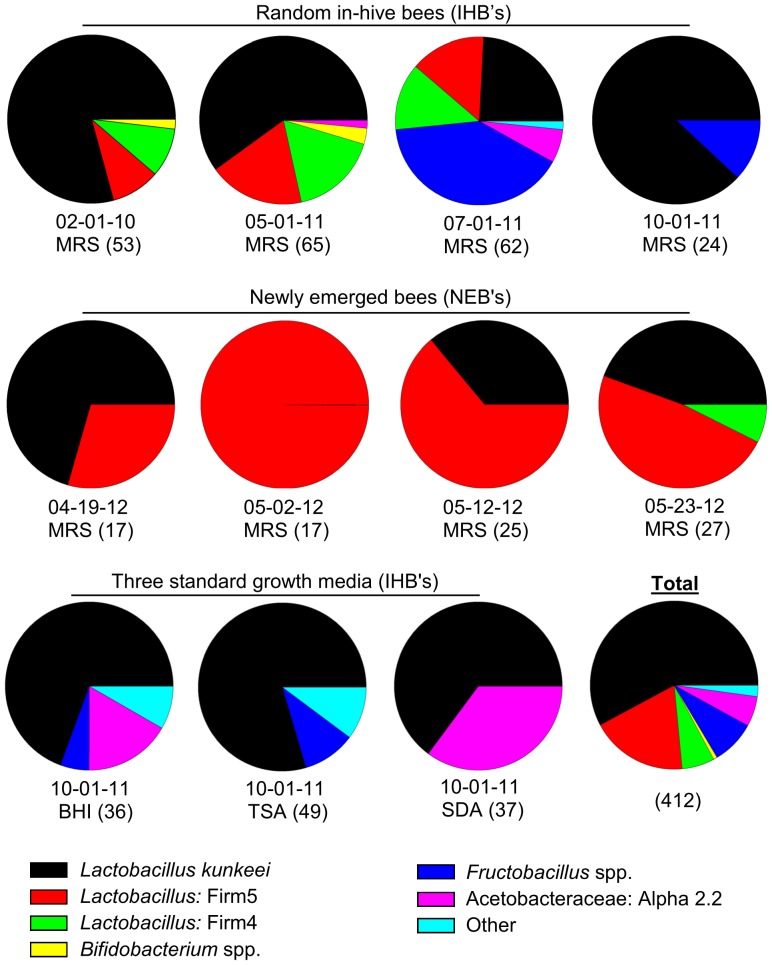
Bacteria cultured from the crop. Pie chart at bottom right shows the proportional grand total. In the upper row, bacteria were cultured on MRS (deMan Rosaga Sharp) media over a yearly time course. The middle row is a month long time-course, sampling only newly emerged bees (NEB's) that had no contact with older siblings, but were allowed to feed on food stores present in the wax comb. The lower row shows culturing results from three standard media types: Blood Heart Infusion; BHI, Sabaroud dextrose agar; SDA, and Tryptic soy agar; TSA. Note that the top right pie chart corresponds to the same time period and pool of sampled individuals as does the lower row. See Table S2 for detailed taxonomy.

We detected within hive transmission of sequences corresponding to core gut bacterial groups. *Lactobacillus* Firm5 was more frequently isolated from the crops of newly emerged bees (NEB) than the crops of random in-hive bees (Fig. 4). The difference between the crop communities was highly significant when considering only those isolates derived from MRS media (Chi-sq  = 65.9, P<0.0001).

### Beebread bacterial clones

**Figure 5:**
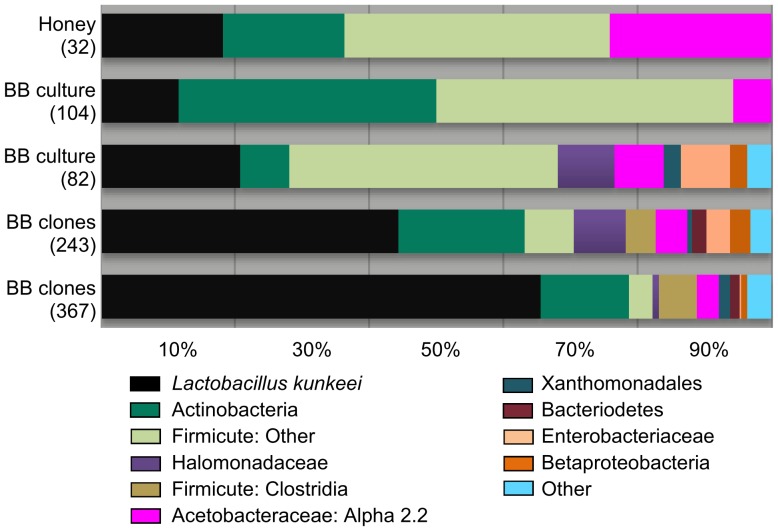
Bacterial communities found in food stores. Communities based on either 16S cloning or sequenced isolates from multiple growth media. Number of sequences for each niche is shown in parentheses. Each bar is an independent sampling event. See Table S4 for detailed taxonomy.

From beebread, we retained 610 cloned sequences following post-quality filtering and removal of chimeras and chloroplast sequences (Table S4, Genbank accession numbers provided following submission). In agreement with culture-dependent results, the most abundant bacterium in both beebread clone libraries was *Lactobacillus kunkeei* (Fig. 5). RDP and BLASTn classifications reveal that only three of the cloned phylotypes had been identified previously as core gut bacteria of honey bees: *Lactobacillus* Firm5, Alpha 2.2 and *Snodgrassella alvi*. Classified by RDP as *Saccharibacter*, the acid-tolerant putative aerobe Alpha 2.2 was abundant in clone libraries from both colonies. Order Actinomycetales was also prevalent, represented primarily by the genera *Corynebacterium*, *Propionibacterium*, and *Rhodococcus* (Table S4). Both colonies revealed an unidentified, perhaps novel genus of uncultured Halomonadaceae, 99% similar to sequences found in Leaf-Cutter Ant fungus gardens, and most related to the genera *Carnimonas* and *Zymobacter*. Many different non-core Firmicutes consisting of obligate or facultative aerobes were also identified (e.g. Clostridia). Although beebread was sampled from neighboring colonies at the same time, their community structure differed exclusive of abundance (unweighted UniFrac score  = 0.7162, p<0.001). The Shannon index of diversity was higher for the isolates than for either of the clone libraries (Table S9).

### Culturing vs. cloning

**Figure 6:**
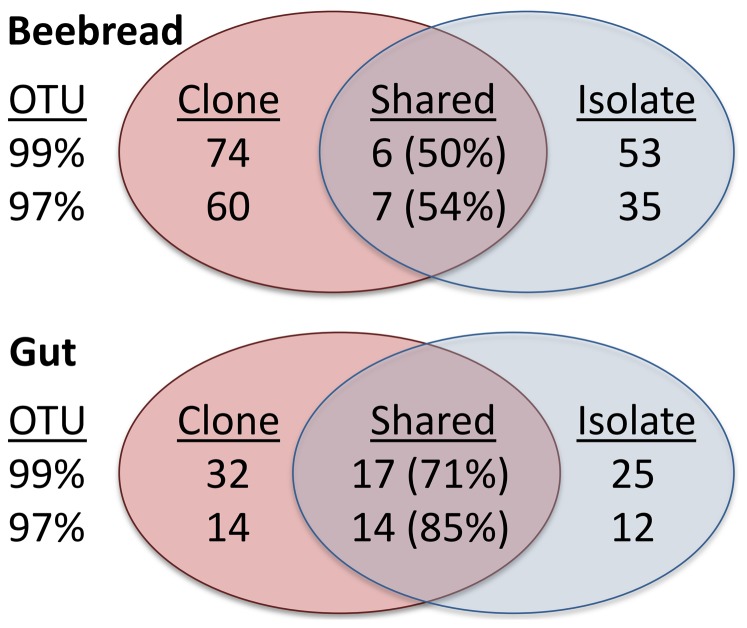
Venn diagrams depicting unique and shared OTU's. Diagrams comparing cultured isolates and cloned sequences derived from beebread and the gut (mid and hind gut). Operational taxonomic units (OTU's) are defined at 99% and 97%. Percent relative abundance of shared OTUs across all libraries is shown in parentheses.

In beebread samples, the total OTU richness for cloning and culturing combined was 102 (97%) and 133 (99%). At 97% and 99% similarity, only 7 and 6 OTU's respectively were shared between beebread clones and isolates (Fig. 6). However, due almost exclusively to the high volume of *L. kunkeei* and Alpha 2.2 sequences revealed by both cloning and culturing, the shared OTU's represented a large proportion of the shared sequences. At 97% and 99% similarity, 53.5% and 49.8% of the total sequences respectively were shared between clones and isolates.

We also compared our sequenced mid and hind-gut isolates to full-length 16S clone sequences derived from DNA extraction specific to the midgut and hindgut of *Apis mellifera* (downloaded from NCBI). Total OTU richness for cloning and culturing combined was 40 (97%) and 74 (99%) OTU's respectively. At 97% and 99% similarity, 14 and 17 OTU's respectively were shared between beebread clones and isolates (Fig. 6). Independent of *L. kunkeei* hindgut abundance, these OTU's more collectively represented a large proportion of the shared sequences. At 97% and 99% similarity, 85.4% and 70.9% of the total sequences respectively were shared between clones and isolates.

### Phylogenetic analysis

Recombination analyses and other data metrics associated with all three phylogenies are presented in Table S6. For the Acetobacteraceae (Alpha 2.2) tree, the pairwise sequence divergence threshold for detecting recombination was not met [56], thus the application of a recombination metric may be invalid. The other two phylogenies are composed of highly divergent families (rather than hypothesized populations, species or genera) of bacteria suggesting that the recombination metric be interpreted with caution (Table S6).

**Figure 7:**
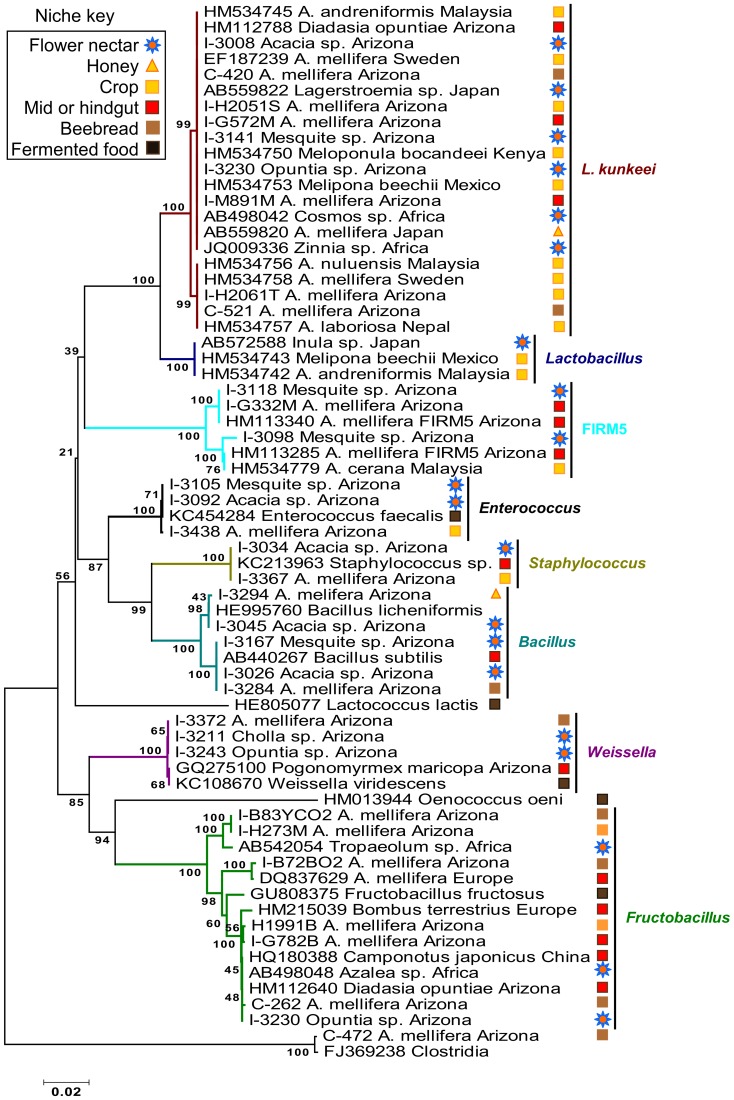
Neighbor joining phylogenetic tree of Firmicutes. Tree based on 1076 positions of the 16S-rDNA bacterial sequence from beebread, honey, alimentary tract and flowers visited by *A. mellifera*, and comparison with related microorganisms (GenBank accessions). Abbreviated taxon labels refer to clones (C), or isolates (I), and symbols mapped to the right of the topology represent the sampled niche. Bootstrap values (n = 1000) are given at the branching points.

The final Firmicute data set contained a total of 67 taxa and 1076 nucleotide positions (Fig. 7). Mapping sampled niche onto the Firmicute phylogeny reveals that the *L. kunkeei* clade is highly invariable, and composed of sequences from diverse habitats and species, including flowers, honey, beebread, and many different species of social and solitary bees from different continents. Found in floral nectar from the immediate pollination environment, many of the 16S sequenced isolates belonging to the Firmicutes share 100% similarity with those found in the guts of adult or larval honey bees, or their food stores. Identical 16S sequences isolated from both flowers and honey bee sources were *Lactobacillus kunkeei, Fructobacillus fructosus*, *Weissella confusa, Staphylococcus* sp., *Bacillus* sp., *Enterococcus* sp. and the core gut bacteria *Lactobacillus* Firm5.

The final Actinomycetyales data set contained a total of 82 taxa and 907 nucleotide positions, and we identified 12 families (Fig. S2). According to results from the RDPII naïve Bayesian classifier, two of the clades formed in the analysis remain undefined at the family level (Table S8). One showed 93% similarity to *Mycobacterium* sp., and the other was 99% similar to an uncultured Actinomycetales clone. With only a couple exceptions, sequences based on isolates and clones were found in completely different parts of this topology. Close bacterial relatives found on GenBank occupy the nests or guts of social Hymenoptera, and most notably, function in cellulose degradation and fungal inhibition.

**Figure 8:**
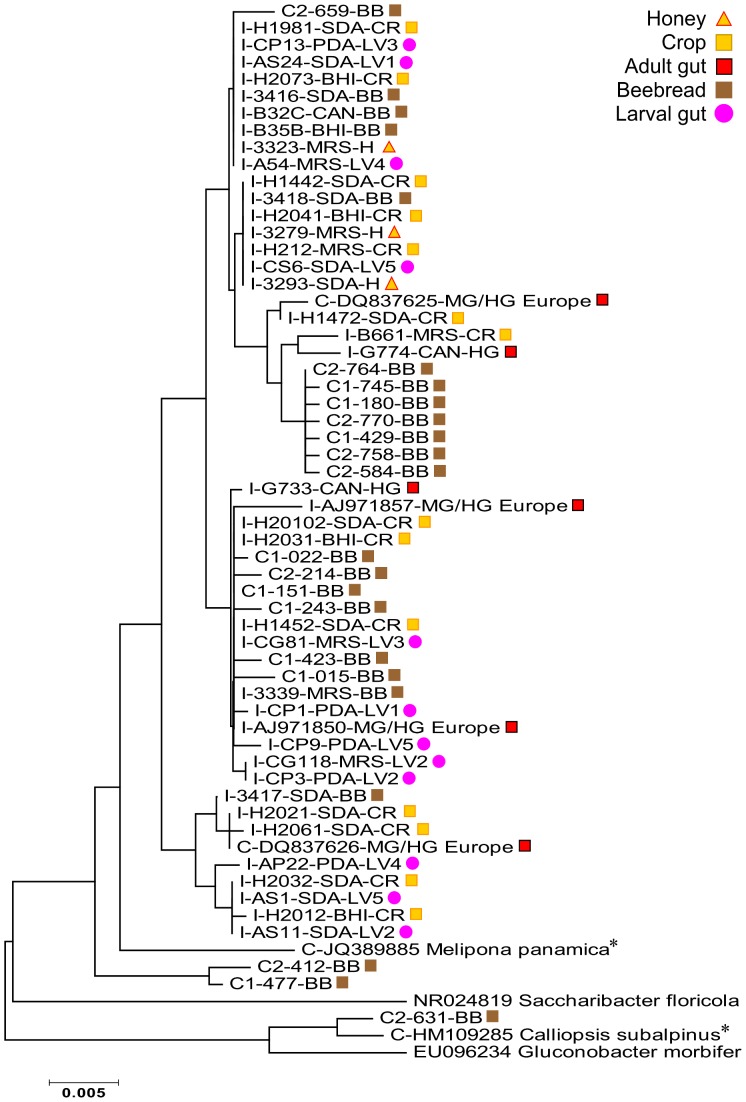
Neighbor-joining phylogenetic tree of Acetobacteraceae (Alpha 2.2). Tree based on 1024 positions of the 16S-rDNA bacterial sequence from a variety of *A. mellifera* associated microenvironments. Sampled niche is mapped to the right of each sequence label (see color key). All mapped sequences are unique according to at least one of the following: sampled niche, culture media, or DNA sequence. Abbreviated taxon labels begin with a letter designating clones downloaded from GenBank (C), clones produced from different colonies (libraries) in this study (C1 = 19, C2 = 20), or isolates (I). Isolates from this study are labeled according to growth media. Isolates from larval guts are according to [82]. Numbers following LV designate the stage of larval instar.

The final Acetobacteraceae (Alpha 2.2) data set contained a total of 62 taxa and 1024 nucleotide positions (Fig. 8). Mapping sampled niche onto the phylogeny indicates that the Alpha 2.2 sequence variation is spread widely throughout beebread, the adult crop and the larval gut. Clones as well as isolates were found throughout the tree, suggesting that much of the Alpha2.2 variability may be cultivable.

## Discussion

Microbial symbioses have been described as a major driver of insect sociality [57]. This point of view invokes consideration of ecological parameters and behavioral traits that may encourage persistent symbioses across the solitary/social insect spectrum [58]. In addition to interactions inherent in life history and social structure are environmental influences, niche construction, nest substrate, and food storage [59]–[61]. In part, these components form the anatomical nest structure that underlies the physiology of the hive [5], [62], [63]. Here we examined pollination and hive microenvironments to investigate the role of microbiology in the honey bee superorganism. Our results reveal that bacteria found in local floral nectar sources are also found frequently in the honey bee hive environment and alimentary tract. Bacteria considered core to the mid or hindgut were easily cultivable, and some could be transmitted via the hive and floral environment. Bacteria from the honey bee foregut (crop) were most similar to those found in the food stores suggesting the inclusion of the crop in the set of structures that influence hive microbial balance.

Our broad survey of nine honey bee-associated microenvironments produced 1723 high quality 16S rRNA gene sequences with an average length >1000 bp. We identified many identical or highly similar 16S sequences found in both culture-dependent and culture-independent studies of the honey bee [24], [26], [33], [38], [44], [64]. A comparison of beta diversity metrics suggest that the sources of variation contributing to our results include sampled microenvironment, culturing bias, and sampling depth (Fig. 1, Table S9). With the exception of Gamma 2, now described as *Frischella parrera* [55], we cultured all of the phylogenetic groups considered core gut bacteria in this analysis (Fig. 2). Within a few of these core groups we cultured considerable diversity consistent with culture-independent surveys (Table S6). Beebread harbored the greatest diversity, but was largely void of core gut bacteria according to both culture-dependent and culture-independent results (Table S4). Despite a greater sampling effort, we found much lower bacterial diversity in the crop as compared to other alimentary tract niches. Despite identical methodology, our broad survey of the crop revealed only 2 of 13 hypothesized sequences (Table S7) labeled as core crop bacteria by other studies [29]–[33]. One of these bacteria (*Lactobacillus* Firm5, strain Bma5) was only found in the crops of newly emerged bees, but was also the most frequent Firm5 phylotype isolated from the hindguts of random in-hive bees. The other was the most abundant isolate from both the crop and the hind-gut, *Lactobacillus kunkeei*.

Our finding that *L. kunkeei* is the most abundant isolate from both the crop and the hindgut is almost certainly due to culturing bias because a variety of culture-independent studies have found little or no *L. kunkeei* in the gut [8], [19], [26], [27]. With cloning or 454-amplicon sequencing of nucleic acids derived from the entire gut or abdomen, phylotypes present at 10^4^ per gram (crop) will not be represented as they constitute less than .01% of the total bacteria in the honey bee alimentary tract. Through repeated culturing of both the crop and hindgut we often resampled *L. kunkeei*, but cloning or next generation sequencing efforts of the entire alimentary tract or whole abdomens rarely detect this bacteria, although the chosen primer sets are an identical match to the *L. kunkeei* sequence [7], [8], [23]–[27], [65], [66]. We suggest that the prevalence of *L. kunkeei* claimed by previous studies [29]–[33] is due in part to culturing bias. In contrast, *L. kunkeei* dominated mature beebread based on both culture-dependent and independent approaches (Fig. 5), suggesting that the abundance of *L. kunkeei* in beebread is real and not an artifact of PCR or culturing bias. Thus, while *L. kunkeei* can be found throughout the alimentary tract, beebread may provide a more stable niche. The 16S rDNA sequence similarity between all honey bee gut segments, 3 of 4 floral sources, and the food stored in the hive was 100% (Fig. 7), suggesting some degree of environmental transmission. Consistent with this finding, *L. kunkeei* is composed of multiple strains [67], [68], and considered the most dominant fructophile in nature, found world-wide in association with many flowers, fruits, and both solitary and social bees [26], [33], [68]–[72]. *Lactobacillus kunkeei* and similar fructophiles (*Fructobacillus* spp.) are often undetectable in the crop or food stores, and their abundance in many bee species is sporadic, seemingly associated with flower type or season [26], [29], [33], [69], [72]–[74]. While highly suggestive of floral transmission or acquisition, many species of bacteria are desiccation tolerant, with the ability to remain in suspended animation for long periods or enter into a viable but non-culturable state [75], [76]. Consistent with this hypothesis, *L. kunkeei* was among the small subset of bacteria revived from pure honey, the most desiccating of hive environments.

### Core crop bacteria

As detailed above, *L. kunkeei* seems to be a “generalist” in fructose rich environments like the honey bee hive and crop. Consistent with our niche predictions for the alimentary tract, the crop was a low diversity, dominance environment, while the midgut and hindgut revealed a much wider variety of both core and non-core bacteria (Tables S2 and S3). Culturing and isolation revealed many *Lactobacillus* Firm4 and Firm5 isolates from the crop that were identical to cloned sequences from the hindgut (Table S7). We found only one of the 13 putative core crop bacteria (strain Bma5), but this bacterium was cultured more frequently from the hind gut than from the crop, and deep sequencing approaches suggest it is abundant in the hindgut [8], [27]. Additionally, strain Bma5 was found only in the crops of newly emerged bees, but not the crops of random in-hive bees, suggesting that it is an ephemeral crop resident captured in the process of establishing in the hindgut (Fig. 4). Thus we find no evidence to support the putative core crop bacterial community [33] but suggest that many bacteria in transit to the hindgut or adapted to the food stores (i.e. *L. kunkeei*, Alpha 2.2) can often be cultured from the crop. Very few bacteria can deal with extreme acid and osmotic stress of the crop, but consistent with our results, pure honey can stimulate the growth of *L. acidophilis* (Firm5) *in vitro*, and strains with striking metabolic resemblance to *L. kunkeei* and Alpha 2.2 have been “rejuvenated” from pure honey [68], [70], [71], [73], [77]–[79]. Thus the crop may serve as a “resuscitation and purification niche” favoring the revival of acid, oxygen, and osmotolerant bacteria harbored in honey or beebread.

A review of the literature reveals no evidence that *Lactobacillus* Firm4, Firm5 and *Bifidobacteria* form a persistent biofilm in the crop. The photograph presented in [33] lacks the stratified nature of a biofilm, and the assay used was designed to stain all bacteria indiscriminately, thus the types of bacteria remain unknown. A more refined approach based on fluorescently labeled and group-specific probes (FISH) found no biofilm in the crop, but did reveal that strains broadly classified as *Lactobacillus* Firm5, *G. apicola* and *S. alvi* (Table 1) are integrated as part of a dense and stratified biofilm in the hindgut [7]. It is likely that core gut bacteria vary concerning their physiological tolerance to the crop and hive environment, but this cannot be distinguished according to variation in the 16S rDNA sequence. While our results provide some perspective concerning the presence of a core crop microbiota, the compendium of available results are consistent with the idea that the majority of *Lactobacillus* Firm4 and Firm5 and *Bifidobacterium* sampled from the crop are transient, and find their primary niche in the hindgut [7], [8], [23], [25]–[27], [69].

### Culturing the core gut bacteria

In contrast to some insect gut environments [80], [81], the cultivable diversity of the honey bee gut is a strong reflection of the taxonomy and diversity found with non-culture based methods (Fig. 1). We cultured representatives from six of seven groups considered core gut bacteria in this analysis (Fig. 2). Relatively low or high sequence diversity relative to abundance may reflect either real diversity, or the inability to represent diversity with culturing (Table S6). In agreement with our culture-dependent results, *Lactobacillus* Firm5 harbors the greatest non-culture based diversity as it is composed of more than one 97% OTU cluster [7]. Based on both culturing and 454-amplicons, *Lactobacillus* Firm4 is also composed of two 97% OTU clusters [8], [33], but extensive culturing efforts suggest that culturing may not adequately represent Firm4 diversity (Table S6). Consistent with their relative absence from the acidic crop and food stores, *Bifidobacteria*, *G. apicola*, *S. alvi* and Alpha 2.1 were sampled almost exclusively from pH neutral media, and were the only core bacteria cultured from the non-acidic midgut (Fig. 2, Table S3). Only a small percentage of our isolates were classified to these four groups, suggesting that neutral media and anaerobic conditions may uncover more cultivable diversity. Although their treatment has been brief in the literature [7], [8], [27], the two commonly occurring groups of Acetobacteraceae (Alpha 2.1 and Alpha 2.2) are apparently very different bacteria based on occupied niche. While Alpha 2.2 is found throughout the hive in beebread, honey, larval guts [82] and crops, our reanalysis of published amplicon libraries agrees with our culture-dependent results, and indicates that Alpha 2.1 is the Acetobacteraceae associated with the adult gut environment.

In contrast to the crop, the hind gut is a stable environment, and much less acidic, receiving a continuous flow of nutrition as simple or modified sugars, partly digested pollen and excreted waste products. In other studied Hymenoptera, the hindgut is the primary niche of actively reproducing bacterial biofilms [83], [84]. According to a recent study, many of the core gut bacteria of the honey bee are concentrated near the hindgut, and *Lactobacillus* Firm4, Firm5, and *Bifidobacterium* are 30–100 times more abundant in the hindgut than the midgut [7]. Our trends of bacterial abundance from each alimentary tract niche are also consistent with other results based on bacterial enumeration of the honey bee gut. While 4–5 log cfu bacteria can be found in the crop, most crop samples contained much less, and many defied bacterial cultivation and/or PCR detection. In contrast, culturing the hindgut is consistently associated with much higher bacterial counts (9–10 log cfu/g) [85], [86].

### Food stores

*L. kunkeei* and Alpha 2.2 were abundant in beebread at all sampling events according to both culture-dependent and independent approaches (Fig. 5). Both were also revived from honey. The taxonomic correspondence among clones and isolates suggests that much of the Alpha 2.2 variability is cultivable (Fig. 8). In contrast, the Actinobacteria found in beebread show virtually no clone/isolate correspondence at the taxonomic level of family (Fig. S2). Other than *L. kunkeei* and Alpha 2.2, very few cloned beebread sequences were represented among beebread isolates and vice versa (Table S8). This methodological difference may be due to multiple factors including growth conditions, primer bias, nucleotide isolation method and seasonal differences. Nevertheless, the extent of the differences still suggests that a complete representation of beebread bacterial diversity may require methodological refinement or the extended application of both culture-dependent and culture-independent methods. In agreement with this point, rarefaction curves on the sampled communities did not asymptote, indicating that much of the diversity remains hidden (Table S9).

Our findings suggest that the beebread fermentation process does not rely on *Lactobacillus* that originates in the gut (Fig. 5). That beebread was dominated by *Lactobacillus kunkeei* suggests that the microbial ecology of beebread may be similar to silage preservation [87], wherein the desired microbial traits are rapid and predominant growth, acid production and tolerance, and the ability to metabolize simple sugars but not organic acids [88]. Our culturing results and the literature indicate that most strains of *L. kunkeei* grow much faster in oxygenated environments, suggesting that nectar and colony food stores rich with hydrogen peroxide or exposed to the air encourage rapid acid production by *L. kunkeei* [70]. Although found at varying frequency, highly aerotolerant bacteria like Alpha 2.2 and some Actinobacteria may also play an important role in preserving beebread, particularly at the interface with oxygen. The strong representation of facultative and obligate anaerobes suggests that beebread becomes anaerobic to some degree after being packed tightly into wax storage cells.

Samples from both clones and cultures demonstrate that Alpha 2.2 was the most common and active Acetobacteraceae in beebread and the crop (Fig 5, Tables S2 and S4). Similar bacteria occur in the pollen provisions of both solitary and social bees, where they are thought to protect both food stores and developing larvae [22], [72], [89]. Highly osmotolerant, gluconic acid producing strains of Acetobacteraceae cultured directly from honey continue growth at 40–50% sugar concentrations and pH 3 [77], [89]. Alpha 2.2 has been found at low frequency in the midgut, but is not commonly found in the increasingly anoxic hindgut [85], where it may be replaced by Alpha 2.1 (Fig. 2). Alpha 2.2 occurs in beebread, larvae and 9 day old bees [7], the age at which bees typically perform nurse duties, consuming beebread to produce highly nutritious larval food with their head glands. Combined with our results, these findings suggests that Alpha 2.2 is not core to the adult gut, but may be best adapted to the crop, honey-rich food stores and the larval gut, and/or associated with brood care or nurse bee hypopharyngeal gland secretions.

An abundance and variety of Actinobacteria occurred in mature beebread (Fig. S2), a result consistent with past microbial investigations of honey bees and other pollinators [23], [74], [90]–[92]. Some Actinobacteria are known plant pathogens, capable of cellulose digestion, but are generally considered protective mutualists in insects, generating secondary metabolites that inhibit fungal growth and deter food spoilage organisms [93]–[97]. The occurrence of similar Actinobacteria groups in the pollen provisions of solitary leaf cutting bees that collect nectar, but don't make honey or wax [91], suggest that some Actinobacteria may be vectored directly from plants or the soil. Actinobacteria similar to those we found in beebread have also been detected in honey bee larva, wax, larval casings, and the midguts of adult bees [23], [74], [92], [98]. Common in bees, many *Strepomyces* spp. produce candicidin [96], an anti-fungal active against a common honey bee yeast. They have also been demonstrated to inhibit bacteria that cause widespread brood disease in honey bees [92]. Represented primarily by the genera *Streptomyces*, *Propionibacterium, Mycobacterium*, and *Corynebacterium*, honey bee associated Actinobacteria may inhibit fungal growth to extend the shelf life of beebread.

### Bacterial transmission

Our findings suggest that at least some of the core gut bacterial strains (Table 1) can survive in the hive environment, facilitating their transmission to the alimentary tract of newly emerged bees (Table S2, S3). Others may rely on direct contact with older siblings [7], [99]. While Alpha 2.2 seems to preferentially occupy or find refuge in beebread and honey, it is also found with considerable frequency in the crop, suggesting that it is adapted to survive host colony division (swarming) to achieve transmission between generations. The niche occurrence and *in-vitro* growth of *Bifidobacterium* suggests both acid and oxygen intolerance. All *Bifidobacterium* were historically considered highly intolerant of oxygen, but honey bee-associated *Bifidobacterium* was recently discovered to utilize oxygen at low levels and harbor genes for respiration and the metabolism of reactive oxygen species [100]. A culture-dependent approach will greatly facilitate the investigation of core bacterial transmission. For example, we detected *Lactobacillus* Firm5 with much lower frequency from the crops of random IHB's than from the crops of NEB's denied contact with older siblings, but allowed contact with the hive environment. Our culturing results suggest that continued application of neutral media may reveal a greater variety of core gut bacteria during transmission (Fig. 3).

Although bacteria evolved to live in the pollination environment may be at a disadvantage in high sugar environments, certain strains of nectar-or flower associated bacteria may be well adapted to the crop and food stores of honey bees. Recent results suggest that floral nectar contains an abundance of unique and diverse bacteria, many of which are highly osmotolerant [101], [102]. While many of these bacteria may be benign, some may have serious implications for honey bee health, by interfering with the establishment of core gut bacteria, or by providing protection from pathogens and preserving food stores [72], [73], [77], [103]–[105]. While a recent study of apple flower microbiome succession did not focus on bacteria associated with pollinators, a quick glimpse of the 454-amplicon data [106] from flowers exposed to honey bees for 3 days revealed a number of core and non-core bacteria of honey bees including core-gut *Lactobacillus*, Actinobacteria, *L. kunkeei* and Alpha 2.2. While the viability of these bacteria are unknown, our study revived *L. kunkeei* from 3 of 4 sampled flowers, the core gut bacteria *Lactobacillus* Firm5 from one flower type, and demonstrated that floral nectar in general was host to a variety of Firmicute related sequences with 100% identity to hive, crop, or honey bee gut samples (Fig. 7). Unsurprisingly, the combined results indicate that many core and non-core bacteria associated with the honey bee may be transmitted with varying frequencies via the pollination environment [99], [106], [107].

### Perspective

The predicted abundance of bacteria in the honey bee hive suggests the potential for many moderately abundant, yet undetected “core-hive” bacteria present at 10^4^–10^5^ bacteria/gram [86], [108]. Both enduring hive bacteria and those continually vectored from the environment could have a broad range of incidence, play major roles in the ecology of the larval, adult or beebread community, or represent a seed bank of species that thrive under different conditions [109], [110]. More broadly, our findings suggest that changes in the pollination environment, due to both typical environmental variation, and human influence could affect ecosystem health by directly or indirectly altering the evolution, abundance, transmission rate or survival of microbes inhabiting floral nectar, the phyllosphere, local water sources or the honey bee hive. As nectar has evolved to perform many of the same antiseptic functions as honey, it is unsurprising that many different microorganisms have evolved to inhabit both flowers and beehives [68], [106].

## Supporting Information

Figure S1 **Neighbor joining phylogenetic tree of Actinomycetales.**
(TIF)

Figure S2 **Bacterial communities in floral nectar.**
(TIF)

Table S1 **Sampling summary and bacterial growth by culture conditions.**
(XLSX)

Table S2 **Accessions, RDP classifications and NCBI hits for crop isolates.**
(XLSX)

Table S3 **Accessions, RDP classifications and NCBI hits for midgut and hindgut isolates.**
(XLSX)

Table S4 **Accessions, RDP classifications and NCBI hits for food store clones and isolates.**
(XLSX)

Table S5 **Accessions, RDP classifications and NCBI hits for flower isolates.**
(XLSX)

Table S6 **Average sequence divergence of isolates and phylogenetic data metrics.**
(XLSX)

Table S7 **Sequence similarity between crop and hindgut isolates.**
(DOCX)

Table S8 **Taxonomic differences between beebread clones and isolates.**
(DOCX)

Table S9 **Alpha and Beta diversity metrics.**
(XLSX)

Text S1 **Culturing microenvironments.**
(DOCX)
