# Supplementary figures and images for: Microbial Ecology of the Hive and Pollination Landscape: Bacterial Associates from Floral Nectar, the Alimentary Tract and Stored Food of Honey Bees (Apis mellifera)
Source: PLoS One. 2013 Dec 17;8(12):e83125. doi: 10.1371/journal.pone.0083125 (PMC3866269; doi:10.1371/journal.pone.0083125)

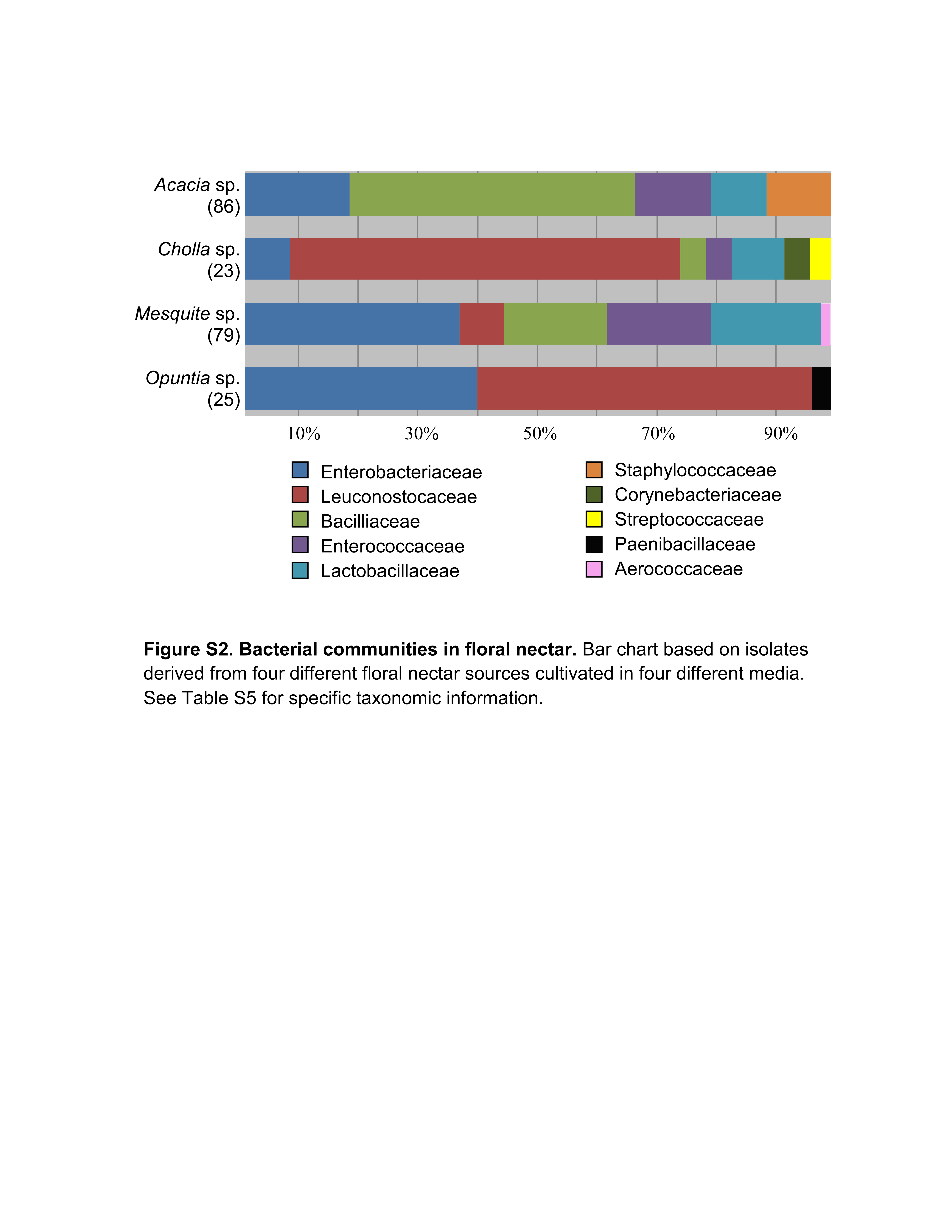

Supplement: Figure S2 — Bacterial communities in floral nectar. (TIF) [file pone.0083125.s002.tif]
