# Supplementary material for: Microbial Ecology of the Hive and Pollination Landscape: Bacterial Associates from Floral Nectar, the Alimentary Tract and Stored Food of Honey Bees (Apis mellifera)
Source: PLoS One. 2013 Dec 17;8(12):e83125. doi: 10.1371/journal.pone.0083125 (PMC3866269; doi:10.1371/journal.pone.0083125)
Supplement: Table S7 — Sequence similarity between crop and hindgut isolates. (DOCX) [file pone.0083125.s009.docx]

Table S7. Bacterial isolates showing ≥99.9% sequence similarity with published GenBank clones and isolates.

| **Sequenced isolates from this study** | | | | **GenBank clones and isolates from other studies** | | | |
| --- | --- | --- | --- | --- | --- | --- | --- |
| **Gut Tissue** | Isolate^‡^ | Ave bp. | Seq. (n) | Phylo group* | Accession# crop^¶^ sample | Accession# gut^†^ sample | Gut tissue^†^ |
| **IHB Hindgut** | |  |  |  |  |  |  |
|  | G5-7-1M | 1135 | 16 | Firm5 | EF187242 | HM111880 | SA |
|  | G3-4-4M | 1207 | 8 | Firm5 | EF187242 | HM113230 | PG |
|  | G5-12-4M | 1207 | 4 | Firm5 | HM534779 | HM113285 | PG |
|  | G3-4-3C | 1207 | 2 | Firm5 | HM534803 | HM046569 | MG |
|  | G3-1-2M | 1145 | 2 | Firm5 | HM534802 | --- | --- |
|  | G7-8-1M | 967 | 2 | Firm5 | --- | HM113350 | PG |
|  | G7-5-2C | 1207 | 2 | Firm5 | --- | HM112071 | SA |
|  | G7-5-1C | 1207 | 1 | Firm5 | --- | HM111925 | SA |
|  | G7-2-3M | 1277 | 2 | Firm4 | --- | HM113271 | PG |
|  | G5-2-2T | 830 | 3 | Bifido | HM534828 | --- | --- |
|  | G7-2-2B | 771 | 1 | Bifido | HM534828 | HM113353 | PG |
|  | G7-4-5B | 1282 | 1 | Bifido | HM534836 | HM112025 | SA |
| **NEB Crop** | |  |  |  |  |  |  |
|  | 5-2-22 | 757 | 39 | Firm5 | EF187242 | AY370183 | SA |
|  | 5-23-12 | 727 | 7 | Firm5 | HM534798 | HM046568 | MG |
|  | 5-2-54 | 757 | 3 | Firm5 | HM534801 | AY667698 | WG |
|  | 4-19-21 | 757 | 1 | Firm5 | --- | HM112080 | SA |
|  | 4-19-6 | 757 | 1 | Firm5 | HM534797 | HM113302 | PG |
|  | 5-23-9 | 692 | 2 | Firm4 | HM534813 | HM113352 | PG |
| **IHB Crop** | |  |  |  |  |  |  |
|  | H6-5-2M | 1051 | 7 | Firm5 | --- | HM113181 | PG |
|  | B6-2-1 | 1053 | 6 | Firm4 | HM534813 | HM113155 | PG |
|  | B7-4-1 | 974 | 4 | Firm5 | HM534804 | HM113337 | PG |
|  | H7-2-3M | 1220 | 3 | Firm5 | --- | HM111983 | SA |
|  | B6-2-1-2 | 1052 | 2 | Firm5 | HQ842700 | HM113348 | PG |
|  | B7-2-2 | 1052 | 1 | Firm5 | --- | HM113340 | PG |
|  | H7-6-6M | 1304 | 1 | Firm5 | --- | HM113343 | PG |
|  | H7-11-1M | 745 | 1 | Firm5 | --- | HM113345 | PG |
|  | H7-11-4M | 1103 | 6 | Firm4 | HM534813 | HM113315 | PG |
|  | H8-5-3M | 1303 | 3 | Firm4 | --- | HM113271 | PG |
|  | H7-10-1M | 1109 | 1 | Bifido | HM534829 | AY370184 | SA |
|  | B7-10-1 | 852 | 1 | Bifido | --- | HM113111 | PG |

Abbreviations; IHB: in-hive bee, NEB: newly emerged bee.

‡ Representative 16S sequence, sequences with unresolved nucleotides were not compared.

* According to [1–4]. Firm4 and Firm5 are distinct *Lactobacillus* clades. Bifido: *Bifidobacterium*

¶ GenBank accessions derived from crop isolates.

† GenBank accessions derived from clones. Single abdomen; SA, 80 pooled guts; PG, whole gut; WG, or mid gut; MG.

1. Babendreier D, Joller D, Romeis J, Bigler F, Widmer F (2007) Bacterial community structures in honeybee intestines and their response to two insecticidal proteins. FEMS Microbiol Ecol 59: 600–610. Available: http://www.ncbi.nlm.nih.gov/pubmed/17381517.

2. Martinson VG, Danforth BN, Minckley RL, Rueppell O, Tingek S, et al. (2011) A simple and distinctive microbiota associated with honey bees and bumble bees. Mol Ecol 20: 619–628. Available: http://www.ncbi.nlm.nih.gov/pubmed/21175905.

3. Cox-foster DL, Conlan S, Holmes EC, Palacios G, Evans JD, et al. (2007) A metagenomic survey of microbes in honey bee colony collapse disorder. Science 318: 283–287. Available: http://www.ncbi.nlm.nih.gov/pubmed/17823314.

4. Moran NA, Hansen AK, Powell JE, Sabree ZL (2012) Distinctive Gut Microbiota of Honey Bees Assessed Using Deep Sampling from Individual Worker Bees. PLoS One 7: e36393. Available: http://dx.plos.org/10.1371/journal.pone.0036393.
