# Supplementary material for: Microbial Ecology of the Hive and Pollination Landscape: Bacterial Associates from Floral Nectar, the Alimentary Tract and Stored Food of Honey Bees (Apis mellifera)
Source: PLoS One. 2013 Dec 17;8(12):e83125. doi: 10.1371/journal.pone.0083125 (PMC3866269; doi:10.1371/journal.pone.0083125)
Supplement: Table S8 — Taxonomic differences between beebread clones and isolates. (DOCX) [file pone.0083125.s010.docx]

Table S8. Bacteria in beebread cultures and clones by 16S rDNA sequence taxonomy and abundance.

| Order | Family | Genus* | Sequence,  Species^†^, or **clade^‡^** | Representative  BLASTn hit | Culture (200) | Clone  (611) |
| --- | --- | --- | --- | --- | --- | --- |
| Actinomycetales | Streptomycetaceae | *Streptomyces* | many species | EU551719 | 0.170 | --- |
|  | Nocardiaceae | *Williamsia* | sp. SY3 | EU073114 | 0.005 | --- |
|  | Pseudonocardiaceae | *Goodfellowiella* | *coeruleoviolacea* | NR043539 | 0.015 | --- |
|  | Microbacteriaceae | *Microbacterium* | *imperiale* | JN585685 | 0.010 | --- |
|  |  | undetermined | clone Plot4-2F07 | JQ359111 | --- | 0.002 |
|  | Micrococcaceae | *Micrococcus* | *luteus* | JN602241 | 0.015 | --- |
|  |  | *Arthrobacter* | sp. Ha21 | JX949306 | 0.005 | --- |
|  | Intrasporangiaceae | *Janibacter* | *sanguinis* | JX435047 | 0.005 |  |
|  | Geodermatophilaceae | *Modestobacter* | *marinus* | FO203431 | 0.005 | --- |
|  | Corynebacteriaceae | *Corynebacterium* | many species | HM205109 | 0.010 | 0.044 |
|  | Propionibacteriaceae | *Propionibacterium* | *acnes* | CP002815 | --- | 0.067 |
|  | Actinomycetaceae | *Actinomyces* | *odontolyticus* | NR041983 | --- | 0.002 |
|  | Nocardiaceae? | undetermined | undetermined | EU825951 | --- | 0.030 |
|  | Kineosporiaceae | *Kineococcus* | *lusitanus* | FN824365 | --- | 0.002 |
|  | undetermined | undetermined | clone nbw1079d02c1 | GQ058336 | --- | 0.002 |
|  |  |  |  |  |  |  |
| Bacillales | Staphylococcaceae | *Staphylococcus* | many species | AJ717375 | 0.125 | 0.016 |
|  | Bacillaceae | *Bacillus* | many species | FN663624 | 0.105 | --- |
|  |  | *Oceanobacillus* | *oncorhynchi* | GU339281 | 0.005 | --- |
|  |  | *Paenibacillus* | *illinoisensis* | AB681007 | 0.015 | --- |
|  | Incertae Sedis | *Gemella* | clone ncd512d12c1 | HM273360 | --- | 0.003 |
|  | Leuconostocaceae | *Weissella* | sp. NBRC 107217 | AB682516 | 0.005 | --- |
|  | Thermoactinomycetaceae | undetermined | bacterium 1335 | JQ798972 | --- | 0.003 |
|  |  |  |  |  |  |  |
| Bacteroidales | Porphyromonadaceae | *Porphyromonas* | clone ncd337d11c1 | HM318418 | --- | 0.010 |
|  | Prevotellaceae | *Prevotella* | *pallens* | AB547703 | --- | 0.006 |
|  |  |  |  |  |  |  |
| Bdellovibrionales^¶^ | Bdellovibrionaceae | *Vampirovibrio* | clone SS-139 | AY945890 | --- | 0.010 |
|  |  |  |  |  |  |  |
| Burkholderiales | Comamonadaceae | undetermined | undetermined | AB491963 | 0.005 | --- |
|  |  |  |  |  |  |  |
| Clostridiales | Incertae Sedis | *Anaerococcus* | many species | HM587319 | --- | 0.016 |
|  |  | *Finegoldia* | *magna* | AB691573 | --- | 0.005 |
|  |  | *Mogibacterium* | clone AF_H06 | AY821870 | --- | 0.003 |
|  |  | *Peptoniphilus* | 375 strain F0436 | HM596293 | --- | 0.013 |
|  | Lachnospiraceae | *Blautia* | clone H1-plate4_H05 | HQ176155 | --- | 0.002 |
|  |  | incertae_sedis | clone ncd1900g03c1 | JF163716 | --- | 0.005 |
|  | Ruminococcaceae | *Faecalibacterium* | clone SJTU_E_09_23 | EF400129 | --- | 0.003 |
|  |  | *Ruminococcus* | clone ncd109c06c1 | HM257639 | --- | 0.003 |
|  |  |  |  |  |  |  |
| Enterobacteriales | Enterobacteriaceae | undetermined | sp. JSC-N3-112-2 | JF958137 | 0.025 | --- |
|  |  | *Enterobacter* | *hormaechei* | HQ202859 | 0.005 | --- |
|  |  | undetermined | clone ncd864f02c1 | HM306316 | --- | 0.003 |
|  |  | *Escherichia/Shigella* | *K-12 substr. MDS42* | AP012306 | --- | 0.003 |
|  |  |  |  |  |  |  |
| Flavobacteriales | Flavobacteriaceae | *Capnocytophaga* | *leadbetteri* | DQ012358 | --- | 0.003 |
|  |  |  |  |  |  |  |
| Fusobacteriales | Leptotrichiaceae | *Leptotrichia* | clone ncd1103b07c1 | HM337602 | --- | 0.002 |
|  |  | *Fusobacterium* | *nucleatum* | FJ471650 | --- | 0.002 |
|  |  |  |  |  |  |  |
| Gp3(Acidobacteria) | Gp3 | *Gp3* | *KBST1R2149270h1* | HM062169 | --- | 0.003 |
|  |  |  |  |  |  |  |
| Lactobacillales | Aerococcaceae | undetermined | undetermined | NR026481 | --- | 0.002 |
|  | Carnobacteriaceae | *Dolosigranulum* | *pigrum* | AB680900 | --- | 0.003 |
|  | Enterococcaceae | *Enterococcus* | *faecalis* | AB362599 | 0.010 | --- |
|  | Lactobacillaceae | *Fructobacillus* | *fructosus* | AB680098 | 0.120 | --- |
|  |  |  | *pseudoficulneus* | NR042758 | 0.020 | --- |
|  |  |  | *durionis* | NR042285 | 0.010 | --- |
|  |  | *Lactobacillus* | *kunkeei* 100-1 | JQ009336 | 0.160 | 0.568 |
|  |  | *Lactobacillus* | **Firm 5^‡^** | HM534813 | 0.005 | 0.002 |
|  |  | *Lactobacillus* | **Firm 4^‡^** | HM113352 | 0.010 | --- |
|  | Streptococcaceae | *Lactococcus* | *raffinolactis* | HM218697 | --- | 0.002 |
|  |  | *Streptococcus* | many species | JF220312 | --- | 0.018 |
|  |  |  |  |  |  |  |
| Neisseriales | Neisseriaceae | undetermined | ***Snodgrassella alvi*^‡^** | AY370189 | 0.005 | 0.002 |
|  |  | *Neisseria* | clone 7H59 | JX010905 | --- | 0.002 |
|  |  | undetermined | undetermined | HM316213 | --- | 0.015 |
|  |  |  |  |  |  |  |
| Oceanospirillales | Halomonadaceae | *Zymobacter* | *palmae* | AB681768 | 0.030 | --- |
|  |  | *Carnimonas* | clone BIGH1426 | HM557913 | --- | 0.039 |
|  |  |  |  |  |  |  |
| Pasteurellales | Pasteurellaceae | *Haemophilus* | clone 069077_015 | JQ448274 | --- | 0.010 |
|  |  |  |  |  |  |  |
| Pseudomonadales | Moraxellaceae | *Acinetobacter* | sp. NF4 | EF565936 | 0.015 | --- |
|  |  |  | sp. DP1 | JN228324 | --- | 0.002 |
|  |  |  |  |  |  |  |
| Rhodospirillales | Acetobacteraceae | undetermined | **Alpha 2.2^‡^** | AJ971850 | 0.060 | 0.033 |
|  |  | undetermined | **Alpha 2.1^‡^** | HM112118 | 0.010 | --- |
|  | Rhodospirillaceae | *Skermanella* | *aerolata* | HQ234263 | 0.005 | --- |
|  |  |  |  |  |  |  |
| Selenomonadales | Veillonellaceae | *Veillonella* | clone ncd1701g12c1 | JF149349 | --- | 0.005 |
|  |  |  |  |  |  |  |
| Sphingobacteriales | Chitinophagaceae | undetermined | clone SC-2_14 | JX574841 | 0.005 | --- |
|  |  | *Hydrotalea* | K6-27 | EF612322 | --- | 0.003 |
|  |  | *Segetibacter* | clone BP20-2E | DQ404676 | --- | 0.002 |
|  |  | undetermined | clone SHNX388 | HM110986 | --- | 0.002 |
|  |  |  |  |  |  |  |
| Sphingomonadales | Sphingomonadaceae | *Novosphingobium* | MG37 | AJ746094 | --- | 0.008 |
|  |  |  |  |  |  |  |
| TM7 | TM7 Incertae Sedis | TM7 Incertae Sedis | clone ncd2718a11c1 | JF235126 | --- | 0.002 |
|  |  |  |  |  |  |  |
| Xanthomonadales | Xanthomonadaceae | undetermined | sp. S6-257 | JQ660206 | 0.010 | --- |
|  |  | *Dyella* | clone DMS34 | FJ536904 | --- | 0.007 |
|  |  | *Stenotrophomonas* | *maltophilia* | CP002986 | --- | 0.005 |
|  |  | *Rhodanobacter* | sp. GR18-2 | FJ821730 | --- | 0.002 |

* Taxonomic ranks for genus level and above according to naïve Bayesian classifier (RDPII) with default parameters (80%). Sequences below 80% for a particular rank are undetermined; sequences undetermined at the Order rank are not shown.

† Sequences or species share >99% similarity to the listed NCBI BLASTn query.

‡ Sequence shares >99% similarity with one or more members of the core gut phylogenetic clades according to [1,2].

¶ Potentially misclassified by RDPII. NCBI BLASTn and SILVA return Cyanobacteria.

1. Martinson VG, Danforth BN, Minckley RL, Rueppell O, Tingek S, et al. (2011) A simple and distinctive microbiota associated with honey bees and bumble bees. Mol Ecol 20: 619–628. Available: http://www.ncbi.nlm.nih.gov/pubmed/21175905.

2. Kwong WK, Moran N a (2012) Cultivation and characterization of the gut symbionts of honey bees and bumble bees: Snodgrassella alvi gen. nov., sp. nov., a member of the Neisseriaceae family of the Betaproteobacteria; and Gilliamella apicola gen. nov., sp. nov., a member of Orbaceae . Int J Syst Evol Microbiol. Available: http://www.ncbi.nlm.nih.gov/pubmed/23041637. Accessed 9 October 2012.
