# Supplementary material for: Microbial Ecology of the Hive and Pollination Landscape: Bacterial Associates from Floral Nectar, the Alimentary Tract and Stored Food of Honey Bees (Apis mellifera)
Source: PLoS One. 2013 Dec 17;8(12):e83125. doi: 10.1371/journal.pone.0083125 (PMC3866269; doi:10.1371/journal.pone.0083125)
Supplement: Text S1 — Culturing microenvironments. (DOCX) [file pone.0083125.s012.docx]

### Microbial ecology of the hive and pollination landscape: Bacterial associates from floral nectar, the alimentary tract and stored food of honey bees (*Apis mellifera*).

Kirk E. Anderson, Timothy H. Sheehan, Brendon M. Mott, Patrick Maes, Lucy Snyder, Melissa R. Schwan, Alexander Walton, Beryl M. Jones, and Vanessa Corby-Harris.

**Text S1. Culturing microenvironments**

*Alimentary tracts-* We sampled a total of 268 alimentary tracts from 24 colonies. Following quality filtering of cultures and sequences, combined crop, midgut and hindgut samples were derived from 208 individual alimentary tracts (Table S1). The midgut, hindgut or crop of each bee was carefully dissected under sterile conditions in a laminar flow biological cabinet. Separated crops, midguts or hindguts were placed in microcentrifuge tubes containing 50-200ul of physiological saline (0.9% w/v NaCl, 0.1% w/v Tween 80, 0.1% w/v Peptone) according to tissue volume, thoroughly macerated with a sterile plastic pestle and vortexed. Immediately following vortexing, the suspended solution was streaked onto agar plates and incubated until growth was observed (Tables S2 and S3).

*Food stores-* To capture the core bacterial diversity of beebread, we sampled five colonies at two time points representing broad differences in the availability of blooming plant species (Table S4). We first cut the tips off of sterile 1000μl filtered pipette tips to fit honey bee cell size, and then pressed cut pipette tips into the pliable beebread stored in the wax comb, resulting in beebread “plugs”. Beebread samples each consisted of 3-6 pooled plugs from various parts of a single wax comb to capture diversity in beebread age. Beebread plugs were pooled by wax comb of origin, suspended in 500ul of sterile physiological saline, and vortexed for 2 minutes to dislodge and homogenize bacteria. The resulting suspensions were immediately streaked out on a variety of growth media (see Table S1). Honey from both capped and uncapped sources was collected into sterile containers, stored on wet ice, and processed the same day. Honey samples (1ml) were suspended in 5ml saline solution, vortexed, immediately streaked onto plates, and incubated until growth was observed.

*Flowers*- We examined the potential for flower to hive bacterial transmission by culturing the floral nectar of flowers in the immediate pollination environment (Table S5). We hypothesize that many of the bacteria adapted to floral nectar can also be found in the honey bee crop or food stores. We first confirmed flower visitation according to blooming plant species, and then randomly sampled flowers where no bees had been observed. We sampled two species of cacti: *Opuntia* and *Cholla*, and two tree species: *Acacia* and *Prosopis* (Table S1). Flowers or catkins (*Prosopis*) were collected in sterile containers of physiological saline, stored briefly on wet ice and processed the day of collection. *Opuntia* and *Cholla* flowers were carefully dissected removing the petals, anther, stamens, and lower portion of the sepals, leaving only the material that enclosed the nectaries. *Prosopis* catkins were processed by cutting florets from the stem material with sterile scissors. *Acacia* flowers were collected whole. Flowers were pooled by species, then separated into four batches for enrichment culturing in four different liquid growth media (MRS, TSA, BHI, and SDA). After three days of growth in aerobic snap-cap tubes, cultures with turbidity were streaked out onto the corresponding agar based growth media for colony isolation.
